# Genetic Predisposition to Persistent Human Papillomavirus-Infection and Virus-Induced Cancers

**DOI:** 10.3390/microorganisms9102092

**Published:** 2021-10-03

**Authors:** Helen Espinoza, Kim T. Ha, Trang T. Pham, J. Luis Espinoza

**Affiliations:** 1 Faculty of Environmental Health, University of Washington, Seattle, WA 98105, USA; helene2@uw.edu; 2 Faculty of Biology, University of Washington, Seattle, WA 98105, USA; kimtha@uw.edu; 3 Department of Biochemistry Techniques, Faculty of Medical Technology, Hai Phong University of Medicine and Pharmacy, Hai Phong 04201, Vietnam; ptttrang@hpmu.edu.vn; 4 Faculty of Health Sciences, Kanazawa University, Kodatsuno 5-11-80, Kanazawa 9200942, Ishikawa, Japan

**Keywords:** gene association studies, head and neck squamous cell carcinomas, human papillomaviruses, virus-induced cancers, genome-wide association studies

## Abstract

Human papillomaviruses (HPVs) are the most common sexually transmitted pathogens worldwide and among the more than 200 identified HPV types, approximately 15 high risk (HR-HPV) types are oncogenic, being strongly associated with the development of cervical cancer, anogenital cancers and an increasing fraction of head and neck squamous cell carcinomas (HNSCC). HPV-associated cervix cancer accounts for 83% of HPV-attributable cancers, and more than two-thirds of those cases occur in developing countries. Despite the high frequency of HPV infections, in most cases, the virus is cleared by the host immune response and only a small proportion of infected individuals develop persistent infections that can result in malignant transformation, indicating that other elements, including biological, genetic and environmental factors may influence the individual susceptibility to HPV-associated cancers. Previous studies have quantified that heritability, in the form of genetic variants, common in the general population, is implicated in nearly 30% of cervical cancers and a large number of studies conducted across various populations have identified genetic variants that appear to be associated with genes that predispose or protect the host to HPV infections thereby affecting individual susceptibility to HPV-associated cancers. In this article, we provide an overview of gene association studies on HPV-associated cancers with emphasis on genome-wide association study (GWAS) that have identified novel genetic factors linked to HPV infection or HPV-associated cancers.

## 1. Introduction

Human papillomaviruses (HPV) comprise a family of DNA viruses that spread through direct contact and cause benign and malignant lesions of the skin and mucosa of the anogenital and upper aero-digestive tract. More than 200 HPV types have been identified so far, and among them, around 40 types are capable of infecting the genital areas of males and females, making HPV the most common sexually transmitted infection worldwide [1,2].

The infection with certain HPV types is strongly associated with the development of cervical cancer and can also cause anogenital cancers (carcinoma of the anus, penis, vulva and vagina), as well as a considerable proportion of head and neck squamous cell carcinomas (HNSCC) [3]. Based on their carcinogenic potential, HPV types have been categorized into high risk-HPVs (HR-HPV), for those strains with known oncogenic potential and low-risk HPVs (LR-HPV), which are not oncogenic. HR-HPVs types include HPV16, 18, 31, 33, 34, 35, 39, 45, 51, 52, 56, 58, 59, 66, 68; and among them, two HPV types (16 and 18) cause more than 70% of cervical cancers and a high proportion of anogenital and oropharyngeal cancers. LR-HPVs include the HPV types 6, 11, 42, 43 and 44, being the LR-HPVs types 6 and 11 responsible for more than 90% of benign genital warts [2,4]. The carcinogenicity of HR-HPV types is rooted to cellular changes induced by the viral oncoproteins E6 and E7, which once integrated into the host genome undermine cell growth regulatory mechanisms driving the malignant transformation process [2]. 

Despite certain HPVs have evolved sophisticated immune evasion strategies to escape the host immune response, in the majority of cases, HPV infections, including those caused by HR-HPVs, cause no symptoms and within six months of exposure the virus is cleared by an effective immune response in around 50% of the infected individuals and become undetectable within two years in more than 90% of cases, however, some infected individuals develop a persistent infection that may result in malignant transformation of the infected tissues [5]. 

Nearly 5% of all cancers occurring worldwide are attributable to HPV, which represents 630,000 new cancer cases per year, making this virus one of the most important infectious causes of cancer [6]. However, the proportion of cancers attributable to HPV differs substantially by world region, likely due to variations in HPV type-specific prevalence, disparity of screening programs and variable genetic predisposition among populations [7]. Thus, whereas the attributable fraction (the proportion of cancer cases that would not have occurred if HPV had been totally absent from the population) of HPV-associated cancers in women ranges from less than 3% in Australia/New Zealand and the USA, in other populations including India and sub-Saharan Africa, this fraction represents more than 20% of cancers [6]. 

The carcinogenic potential of HR-HPVs has been demonstrated by clinical/epidemiological and molecular studies, however, despite the extremely high frequency of HPV infections, where almost all sexually active adults becoming infected at some point of their lives with at least an HPV type, most infected individuals never develop cancer, suggesting that for the development of HPV-associated cancers, additional factors are necessary. In this regard, several factors, including biological, genetic, environmental factors and other individual features have been investigated for their possible implication in the carcinogenic process triggered by HPVs [8,9]. Accumulating evidence indicate that the coinfection with other microorganisms modulates individual predisposition to HPV-induced carcinogenesis as shown by the increased incidence of premalignant lesions and cervical cancer caused by HPV in individuals infected with human immunodeficiency virus (HIV) [10], as well as in HPV+ women infected with herpes simplex virus type 2 (HSV2) [11], and *Chlamydia trachomatis* [12]. Similarly, alterations in the microbiota composition (dysbiosis) in the HPV-infected tissues appear to predispose to malignant transformation [2,13]. 

The potential impact of genetic factors in the susceptibility to HPV-associated cancers has been extensively studied across different populations using gene association studies via either targeted-gene association studies or genome-wide association study (GWAS). A large study conducted in Sweden estimated that the proportion of cervical cancer susceptibility due to host genetic factors was 27% (8) and this can be mostly attributable to genetic variants common in the general population (9,10). Subsequent, GWAS performed in individuals of European-ancestry and East Asian populations have found statistically significant associations in the HLA locus and outside of it with susceptibility to cervical cancer and HNSCC [14,15,16,17,18] and one GWAS identified genetic risk factors for developing persistent infection with HR- HPVs [19]. 

In addition, small-size studies have also identified single-nucleotide polymorphism (SNP) allele or genotypes of genes involved in the immune response and other biological cell processes, such as cell proliferation, apoptosis, DNA repair, etc. associated with susceptibility to HPV-induced cancers, however, interpreting the implications from some of these studies is challenging due to the enormous amount of data derived from multiple and heterogeneous studies, with some of them reporting contradictory findings, making it difficult to replicate those findings across different populations, and although some of the findings that were unclear from individual study reports have been confirmed by various meta-analysis, more reproducible findings have been revealed by GWASs. Here, we provide an updated overview of gene association studies on HPV-associated cancers (cervical cancer, HNSCC and anogenital cancers) with emphasis on data provided from recent GWASs that have identified novel genetic factors linked to HPV infection or HPV-associated cancers. 

## 2. Candidate Genes versus Genome-Wide Associations in HPV Research

Most gene association studies are carried out using either the candidate gene approach or based on GWASs, in which genetic variants spanning the entire genome of a defined region are tested. Both approaches have a combination of benefits and disadvantages. In general, while candidate gene studies tend to have a higher statistical power, they are incapable of discovering new genes or gene combinations [20]. Candidate gene studies limit the analysis to one or few genes focusing on genetic variations associated with the disease, based on a previously formulated hypothesis about the role of a selected gene, and thus these studies can be biased toward genes and biological pathways related to the researcher’s scientific interest [21]. These studies are typically structured as case-control studies, where cases and controls are first identified, and the genetic differences between the two groups are then determined. In contrast with candidate gene studies, which are hypothesis-guided, GWASs constitute a hypothesis-free approach that simultaneously examines multiple genetic variations across the genome of different individuals to identify candidate genes or genome regions that contribute to a specific disease. This unbiased approach has successfully discovered a number of genetic loci associated with susceptibility to complex diseases, including cancer, autoimmune disorders and certain infections and has opened a new avenue for studying cervical cancer, which could lead to the previously unsuspected discoveries of susceptibility or resistance genes. GWASs can identify new gene variants regardless of whether their function was known before, and although they tend to have a low power owing to the number of independent tests performed, this apparent weakness is less evident when larger sample sizes are analyzed and compared with candidate gene studies, the results from GWASs tend to be more reproducible over time [22]. 

**Figure 1:**
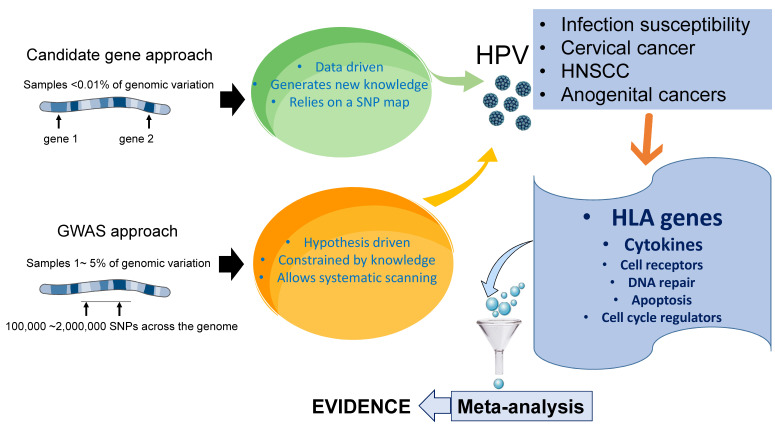
Gene association studies in HPV research.

It is important to mention that due to their affordable cost and technical simplicity, the vast majority of gene association studies in HPV-associated malignancies have been carried out using the candidate gene approach (Figure 1). 

Most gene association studies to identify individual predisposition HPV-associated cancers have been carried out using the candidate gene approach, which limits the analysis to one or few genes, based on a previously formulated hypothesis about the role of a selected gene. These studies are typically structured as case-control studies, where cases and controls are first identified, and the genetic differences between the two groups are then determined. In contrast with candidate gene studies, genome-wide association studies (GWASs) constitute an unbiased hypothesis-free approach that simultaneously examines multiple genetic variations across the genome of different individuals to identify candidate genes or genome regions that contribute to a specific disease. Both, candidate gene studies and GWASs have identified SNPs in genes associated with the immune response, apoptosis, DNA repair, as well as within the HLA region, which appear to predict susceptibility to persistent HPV infection and HPV-induced cancers. Among them, allelic variation within the HLA region is the most consistently identified genetic risk factor. Meta-analyses of these studies have also contributed to verify the reproducibility of the findings.

## 3. Genetic Variants and Susceptibility to HPV Infections

The infectious and replication cycle of HPV is conveniently synchronized to the epithelial differentiation program of the host and consequently, there are no cytolysis or cytopathic changes during the virus replication. After entering the epithelium via skin microlesions, the virus infects immature basal keratinocytes and infected cells migrate to the upper layers of the epithelium carrying the viral genomes and the amplification of the HPV genome and the subsequent production of infectious virions occur once the infected keratinocytes cells undergo terminal differentiation [5,23]. The time from virus entry to release of progeny virions is approximately three weeks, which is equivalent to the time required for the basal keratinocytes to undergo terminal differentiation and desquamation on the surface of the skin and since the virus-infected keratinocytes naturally die during desquamation, there are no major danger signals in the host cells to generate an inflammatory response [2,5]. 

Genetic association studies test for a correlation between the presence of a disease or condition and genetic variation to identify candidate genes or genome regions that influence individual susceptibility to such disease. In the case of HPV infection, since a large number of HPV infected individuals never develop symptoms, it is difficult to accurately determine that a given SNP allele or genotype in a fraction of cases (HPV-infected subjects) and controls (individuals without the infection) increases the risk of an HPV infection.

HLA is a complex of highly polymorphic genes on human chromosome 6 which encode cell-surface proteins that play a critical role in immune regulation. HLA variants have been linked to several human disorders ranging from autoimmune diseases, infections, degenerative disorders and cancer [24]. Since HLA molecules present foreign antigens and promote immune recognition, thus playing a critical role in the clearance of infected cells, polymorphisms in HLA genes may encode proteins with distinct binding affinities (lower or higher) to HPV antigens, thus modulating the recognition of HPV infections, subsequently affecting the individuals’ susceptibility to developing persistent infections [8]. Not surprisingly, the impact of genetic variants in HLA genes has been subjected to multiple gene association studies, however, most studies have focused on the associations between HLA and HPV-induced malignancies, especially cervical cancer and only a few studies have studied the association of *HLA* genes with HPV infections [8,9]. 

A study that enrolled patients from 13 U.S. cities evaluated associations between three SNPs in the interleukin 10 (IL10) promoter and clearance of low- or high-risk HPV infection in a cohort of 226 adolescent females. The GCC haplotype in the *IL10* promoter was associated with reduced clearance of high-risk HPV16-like, HPV18-like and any high-risk type, among immunosuppressed individuals (HIV-1 seropositive and CD4+ ≤500), but not with low-risk HPV type [25]. Given the reported higher production of IL10 associated with the *IL10* GCC haplotype [26], these results suggested that *IL10* variants influence the clearance of infection with high-risk HPV types because higher levels of IL10 may impair the production of inflammatory cytokines such as IL-2, TNF-α, IL-4, IL-6 and IL-12 that are involved in the TH1-TH2 immunoregulation and immunity against HPVs. 

In a case-control study conducted in Brazil, (161 cases and 257 controls), women with no HPV-associated cancer harboring the alleles DRB1*1503, DRB1*0405 and DQB1*0602 were more likely to have an HPV positive test [27] and another study conducted in Mexico (172 women) observed that the allele HLA-DRB1*07 was associated with viral clearance. Conversely, the allele HLA-DQB1*0501 was linked to higher susceptibility to reinfection with HPV and the allele HLA-DRB1*14 was a possible protective factor for the development of cervical cancer [28]. 

In one study conducted in Canada (541 women), the allele *HLA-G(∗)01:01:01* was associated with an increased risk of alpha groups 1 and 3 (being alpha group 1 LR-HPV cervical species; group 2 HR-HPV cervical species; and group 3 LR-HPV vaginal species) and the genotype *HLA-G(∗)01:04:01* was associated with a decreased risk of alpha group 3 infection, however, no allele or genotype associated with HPV persistence [29]. Similarly, another study from Canada examined the impact of *HLA-E* and *HLA-G* polymorphisms HPV infection susceptibility and persistence in 636 female university students in Montreal. Persistent infections with HPV-16 and HPV types from α species 2, 3, 4 and 15 were more commonly observed among women harboring HLA-G*01:01:02. Conversely, *HLA-E* variants were not associated with the risk of acquisition or persistence of HPV infection [30].

A recent study aimed to ascertain the effect of genetic variation on *HLA-DRB1* and *DQB1 HLA-* alleles related to the clearance of six HPV types (HR-HPV-16, -18, -31, -33, -45 and -58) in a cohort of 276 Colombian women observed that HLA allele/haplotype relationship with the clearance of HPV infection correlated with the infecting HPV type, in line with the specific viral epitopes displayed and thus, while DRB1*12:01:01G favored the clearance of HPV-16 and HPV-45, it hindered the HPV-18, HPV-31 and HPV-58 elimination [31]. 

Another study from Nigeria that included 517 individuals, alleles DQA1*01:02 and DQA1*02:01 were positively associated with prevalent but not persistent HR-HPV infections and four haplotypes (A*30:01-DQA1*05:01, B*07:02-C*07:02, B*07:02-DQA1*05:01 and C*07:02-DQA1*05:01) were found in strong association with prevalent cervical HR-HPV infections and several haplotypes that included the DQA1*05:01 allelic variant was significantly associated with persistent cervical HR-HPV infections [32]. The same group also conducted the first GWAS to identify variants associated with cervical HR-HPV infection and persistence and observed that although no single variant reached genome-wide significance it generated suggestive candidate risk loci for cervical HR-HPV infection and persistence, where the top three variants associated with HR-HPV infections included intronic variants clustered in *KLF12* (Kruppel Like Factor 12) and key variants associated with HR-HPV persistence were in gene regions *DAP* (Death Associated Protein), *NR5A2* (Nuclear Receptor Subfamily 5 Group A Member 2), and *MIR365-2* (microRNA 365-2) [19]. Of note, the gene product of *KLF12* (protein KLF12) plays important roles in differentiation, function and homeostasis of dendritic cells, B lymphocytes, natural killer (NK) cells and various T subsets [33], which are critical players in the immune response against HPV infection, including recognizing and eliminating infected cells [2], and molecular studies using the HPV+ cancer cell lines (SiHa and Hela cells) to elucidate the mechanisms of HPV integration in the human genome, showed that *KLF12* was one of the main integration sites for HPV [34], thus indicating that KLF12 may be implicated in the carcinogenesis process triggered by HR-HPV infection.

A multinational case-control study (Mexico, U.S and Brazil) assessed HPV clearance in 40 men (cases) with persistent genital HPV16 infection (>18 months) and 151 controls who were HPV 16-positive, but whose infections cleared in <18 months. Using a genome-wide genotyping platform the variants *rs1293153* and *rs405103*, in chromosomes 20 and 15, respectively, showed the strongest association with genital HPV 16 persistence. Other variants associated with increased risk of HPV16 persistence were located in *SLC12A6, DCC* and *CSMD1* genes [35]. The variant *rs11874458*, located in the intron of the *DCC* (Deleted in Colorectal Cancer) gene which is a tumor suppressor that has emerged as a potential marker of precancerous lesions of the cervix [36], also correlated with persistent HPV16 infection in this study [35]. Similarly, the variant *rs1482207* in *CSMD1* (CUB And Sushi Multiple Domains 1) gene, another tumor suppressor gene that is frequently deleted in several cancers including HNSCC, and carcinoma of the ovary, breast and prostate [37], was also associated with persistent HPV16 infection in this study. On the other hand, the SNP *rs7176426* in the *SLC12A6* gene was associated with clearance of HPV 16 in this study [35]. 

The available evidence indicates that although most gene association studies searching for factors associated with susceptibility to acute or persistent HPV infection have focused on HLA molecules, the application of high throughput technologies, such as GWASs have allowed to identifying not only new genetic factors that predispose to HPV infection but also have contributed to discovering additional elements that appear to be implicated in the pathogenesis of HPV infection and HPV-induced carcinogenesis. 

## 4. Genetic Variants and HPV-Induced Cervical Cancer

Persistent infection with oncogenic HR-HPV subtypes is a necessary factor in the carcinogenesis process associated with cervical cancer, however, despite the high frequency of HPV infections (lifetime incidence over 70%), only around 1% of women infected with cervical HPVs progress to cervical cancer [38]. Various factors have been reported to influence HPV carcinogenesis by affecting either HPV clearance or the risk of progression to cervical cancer. For example, the viral load and repeated HPV infections, as well as the HPV genotype and HPV epigenetic variation are viral features that influence HPV-induced carcinogenesis. Host immune response to infection, tobacco smoking, coinfection with other pathogens, hormonal contraceptives and socioeconomic factors have been also reported to modulate the risk of developing cervical cancer [2,39,40]. In addition, host genetics is an important determinant of HPV-associated cervical cancer with a genetic contribution of 27% according to epidemiological studies, and 36% of liability due to common genetic variants in the population [41]. 

In the past three decades, a large number of candidate gene studies for identifying genes conferring susceptibility to cervical cancer have been reported. Genetic variants in the HLA region have been extensively studied in gene association studies of HPV neoplasm and also the strongest associations with cervical neoplasia are with HLA genes, where both risk and protective alleles have been identified [8,42,43,44]. A recent meta-analysis of 36 case-control studies (6645 cases and 9095 controls), revealed multiple *HLA-DRB1* alleles associated with cervical cancer in women of diverse ancestry populations. Among them, the DRB1*09 and DRB1 *15 were associated with cervical cancer risk and DRB1*13 exerted a protective effect. Conversely, *HLA-DRB1* specific alleles, including DRB1*04:01, DRB1*10:01, DRB1*11:01, DRB1*15:01 and DRB1*15:02 were associated with an increased risk of cervical cancer [45]. Consistent with these observations, in a meta-analysis of 11 studies including 5008 cases and 9322 controls, the variants *HLA-DPB1⁎03:01, DPB1⁎04:02, DPB1⁎13:01*, *rs9277535* and *rs3117027* in the *HLA-DP* gene were significantly associated with cervical cancer [46], thus substantiating the associations between genetic variations in the HLA locus and susceptibility to cervical cancer.

In addition, genetic predisposition has been found outside the HLA region, in genes related to immune response [47], cell cycle control, DNA repair, apoptosis regulation, cell surface receptors and other elements likely involved in carcinogenic pathways. Thus, numerous genetic polymorphisms in immune response genes, including *IL-1* [48], *IL-10* [49], *IL-17* [50], toll-like receptor (*TLRs*) [51], interferon-gamma (*IFN-γ*) [52], tumor necrosis factor-alpha (*TNF-α*) [49,53,54,55] and a variety of genes [55,56,57] have been reported in association with cervical cancer susceptibility, however, inconsistent results are frequently found and most of these findings have not been replicated in larger cohorts. 

IL-10 is a cytokine with potent anti-inflammatory properties that plays a central role in maintaining tissue homeostasis and limiting host immune response to pathogens, however, an excess of IL-10 may impair the function of key immune cells that play a crucial role in the clearance of infectious agents [58]. Within the promoter region of the *IL-10* gene, the three common SNPs, − 1082 A/G (*rs1800896*), − 819 T/C (*rs1800871*) and − 592 A/C (*rs1800872*) have been reported to regulate *IL-10* transcription and expression [59,60], where the GCC haplotype associates with higher production of IL10 [26]. Several candidate gene studies have reported an association to exist between *IL-10* promoter polymorphisms and cervical cancer susceptibility. Thus, women harboring the higher IL-10 production variant have an increased risk of cervical cancer and premalignant lesions, which has been verified across several populations [61,62,63,64,65,66]. These observations have been also confirmed in meta-analyses of not only cervical cancer [67] but also in head and neck cancer [68], indicating that IL-10 plays a crucial role in the host immune response to HPV.

MicroRNAs (miRNAs) are a family of endogenous small RNAs that regulate gene expression thereby regulating the expression of the protein. These non-coding elements affect a wide array of biological processes including carcinogenesis, where they may function as either oncogenes or tumor suppressors [69]. SNPs in miRNA can occur either in the miRNA genes or in the miRNA binding site to target mRNA, which can interfere with the regulatory function of miRNA [70]. Polymorphisms in several miRNAs have been reported in association with HPV persistence and with the development of premalignant lesions and cervical cancer [71,72,73,74]. A recent meta-analysis of six case-control studies testing the effects of SNPs in microRNAs identified the variant *rs531564* (miR-124) in association with an increased risk of cervical cancer [75]. Given that the majority of the genes in the human genome could be regulated by miRNAs [76], it is expected that SNPs affecting microRNA-binding sites in these regions, may have a broad impact on disease pathogenesis, including on the host immune response to HPV. Hence, it is expected that using high-throughput genotyping technologies for the analysis of SNPs related to miRNAs, such as GWASs coupled with machine learning and other artificial intelligence tools, will contribute to better understand the individual predisposition to HPV-associated cancers. 

A literature search of GWASs of cervical cancer identified a total of seven studies (including precancerous and invasive cervical cancer) (Table 1).

Even though those studies varied in size and some discrepant findings were reported, the most consistent allelic variation identified in these GWASs occurred within the HLA region, especially at the 6p21.3 locus. A GWAS in women of European-ancestry, including in 1075 cervical cancer cases and 4014 controls and replicated it in 1140 case subjects and 1058 control subjects identified three independent loci in the MHC region at 6p21.3 in association with cervical cancer, including the variant *rs2516448,* adjacent to the MHC class I polypeptide-related sequence A gene (*MICA*); the *rs9272143*, between *HLA-DRB1* and *HLA-DQA1*, and the *rs3117027*, at *HLA-DPB2*. The study also confirmed previously reported associations of B*0702 and DRB1*1501-DQB1*0602 with susceptibility to and DRB1*1301-DQA1*0103-DQB1*0603 with protection against cervical cancer. The risk allele of *rs2516448* was in linkage disequilibrium with a frameshift mutation of *MICA* gene encoding a truncated MICA protein and complementary molecular studies showed that women carrying this mutation have lower levels of membrane-bound MICA [18]. MICA is a membrane-bound protein that is upregulated in cells exposed to stress stimuli, such as viral infection and malignant transformation, and is recognized by immune cells expressing the activator receptor NKG2D. Immune cells expressing NKG2D, such as NK cells and various T-cell subsets, eliminate target cells expressing MICA and other NKG2D ligands, and thus the NKG2D/MICA axis has been implicated in tumor immunosurveillance [77]. 

A pooled analysis of data from the previous European GWASs and validated in an independent cohort of 961 patients (827 with CIN3 and 123 with cervical cancer) and 1725 controls of European ancestry supported for previously identified loci at 6p21.3 (*rs9271898* and *rs3130196*) and also confirmed associations with reported HLA alleles including HLA-B*07:02, -B*15:01, -DRB1*13:01, -DRB1*15:01, -DQA1*01:03, -DQB1*06:03 and -DQB1*06:02. In addition, the study identified and subsequently replicated an independent signal at *rs73730372* at 6p21.3 (*HLA-DQA1* and *HLA-DQB1*) in association with CIN3 [78]. Another study conducted in Europe, including a total of 2866 cervical cancer cases and 6481 controls confirmed the strong association of the MHC with cervical neoplasia, and identified the amino acid positions 13 and 71 in *HLA-DRB1* and *156 in HLA-B* (within both HLA Class I and Class II alleles). Specifically, three haplotypes, HLA-DRB1*15/HLA-DQB1*0602/HLA-DQA1*0102, HLA-B*0702/HLA-C*0702 and HLA-DRB1*0401/HLA-DQA1*0301, were associated with increased risk of both HPV16 and HPV18-associated cervical cancer, and for the development of both squamous cell carcinoma and adenocarcinoma. Notably, the genotype HLA-B*15 (HLA Class I) was strongly associated with reduced risk of squamous cell carcinoma and HPV16-associated cervical cancer but was not linked to HPV18-associated cervical cancer. No other associations outside the MHC locus were identified [41]. The strong common variant heritability shown in this study indicates that host genetic variation is an important determinant of the development of cervical neoplasia in HPV-affected women.

A GWAS from China that included a discovery set of 1364 women with cervical cancer (cases) and 3028 female controls, revealed an association between *HLA-DPB1* and *HLA-DPB2 (HLA-DPB1/2*) at 6p21.32 and cervical cancer (*rs4282438*) and also identified strong evidence of associations between cervical cancer and two new loci: 4q12 (*rs13117307*), in an intronic region of *EXOC1*, and 17q12 (*rs8067378*) in the *GSDMB* gene [79]. The protein product of *EXOC1* contributes to the formation of the exocyst complex, which facilitates the vesicle transport, secretion and cellular migration and appears to influence the immune response to viral infection [80] and has been implicated in the progression of human oral squamous cell carcinoma [81]. Importantly, a target gene study conducted in the Polish Caucasian population, including 485 women with cervical cancer and 509 controls, corroborated the association between the *rs13117307* SNP with the risk of development and spreading of cervical cancer, and functional assays reported a role of this gene variant regulating *EXCO1* transcription [82]. Gasdermin, the product of the *GSDMB* gene, can induce membrane pores and disrupt cell membrane integrity to trigger inflammatory cell death (pyroptosis) and the release of cellular contents, including inflammatory cytokines into the extracellular space, thus amplifying tissue inflammation and the recruitment of immune cells to the site of infection or damage [83]. 

In a relatively small GWAS involving 226 women with cervical cancer and 186 controls no statistically significant associations were found between the tested SNPs and susceptibility to cervical cancer [84]. A Japanese GWAS of cervical cancer in East Asian populations involving 2609 cases and 4712 controls, which comprised of 1289 cases and 2718 controls from the previously reported Chinese study [79] and two newly reported Japanese studies (1320 cases, 1994 controls), corroborated two previously reported loci at *rs115137622* (downstream *HLA-DPB2*) and *rs8067378* (17q12) in association with cervical cancer and identified significant associations at 5q14 with SNP *rs59661306* and at 7p11 with the SNP *rs7457728*. Notably, in 5q14, the chromatin region of the GWAS-significant SNPs was found to be in contact with the promoter of the *ARRDC3* (arrestin domain-containing 3) gene. Further functional studies showed that *ARRDC3* knockdown in the HPV+ HeLa cells resulted in substantial reductions in cell growth and decreased susceptibility to HPV16 pseudovirion infection [15], indicating a potential role of ARRDC3 in the entry of HPV into the cell, suggesting that genetic variation in *ARRDC3* might affect individual susceptibility to HPV. 

A very recent study (the largest GWAS conducted so far) provided evidence suggesting the presence of a disruption in apoptotic and immune function pathways in association with genetic susceptibility to cervical cancer. This study included a cohort of 273,377 women (4769 CIN3 and invasive cervical cancer case samples and 145,545 control) of unrelated European individuals using data from UK Biobank, and a replication cohort of 128,123 women (1648 invasive cervical cancer cases) from a Finnish dataset. After assessing more than 9 million SNPs, the study found evidence for a strong association at six independent loci with CIN3 and invasive cervical cancer. This included two novel loci, *rs10175462* in the *PAX8* gene and *rs27069* in the *CLPTM1L* gene, and previously reported variants, the SNPs *rs9272050* (*HLA-DQA1*), *rs6938453* (*MICA*), rs55986091 (HLA-DQB1) and *rs9266183* (*HLA-B*). Three of these SNPs, *PAX8* (*rs10175462*), *CLPTM1L* (*rs27069*) and *HLA-DQA1* (*rs9272050*) were replicated in the independent Finnish dataset of 1648 invasive cervical cancer cases. Further Mendelian randomization approach substantiated the risk-increasing effect of smoking, and the number of sexual partners as additional risk factors for developing cervical cancer, and a protective effect of older age at first pregnancy on cervical cancer, thus supporting the role of gene-environment interactions in HPV-associated cancers [14]. 

## 5. Genetic Variants and HPV-Induced Head and Neck Cancer 

Head and neck cell squamous carcinoma (HNSCC) is the sixth most common cancer worldwide with 890,000 new cases worldwide and 450,000 deaths reported in 2018; the incidence of HNSCC is predicted to increase by 30% by 2030 (9). HNSCC includes a variety of malignancies affecting the upper aerodigestive tract, such as oral cavity, oropharynx, hypopharynx and larynx, and most commonly affects male older individuals with heavy tobacco and/or alcohol use history, where the chronic inflammation and the prolonged exposure to carcinogens may result in the accumulation of genetic alterations in key regulatory genes such as *TP53, CDKN2A, NOTCH1, CCND1*, etc. which increases the risk of HNSCC [85]. Even though the use of tobacco and alcohol constitutes the most significant risk factors of HNSCC, the fact that only a fraction of these individuals will develop HNSCC despite the high prevalence of tobacco and alcohol use in the general population suggests that other factors, including genetic predisposition and environmental factors, are also involved. It is estimated that nearly 25% of HNSCC cases occurring worldwide are associated with HR-HPV infection, however, these percentages vary considerably according to geographic factors and also depending on the anatomic site. For example, the HPV prevalence in oropharyngeal cancer appears to be higher than in other HNSCCs, with reports of up to 72% HPV positivity in these tumors [85,86]. 

Similar to cervical cancer, most gene association studies in HNSCC have been carried out using the candidate gene approach and a several SNPs in genes linked to apoptosis, cell cycle regulation, DNA repair and immune response have been reported in association with susceptibility to HNSCC. Through our research, we have found seven GWASs on HNSCC, which have identified genetic variants in the HLA region and in genes outside the HLA (Table 2). 

The oncogenic properties of HPV 16 and 18 are based on the expression of the viral E6 and E7 oncoproteins. While E7 protein binds and inactivates the tumor suppressor Rb, the E6 protein binds to the cellular p53 protein leading to its inactivation or degradation. A functional SNP in exon 4 of the *TP53* gene (p.Arg72Pro) influences the E6 protein-guided degradation [2]. The p.Arg72 variant is more sensitive to the E6 protein guided degradation than the p.72Pro variant, thus cells harboring the p.Pro72 variant are more resistant to apoptotic stimuli due to the reduced traffic of this variant to the mitochondria. A meta-analysis of 13 studies involving 5614 participants (2413 cases and 3201 controls) found, a significant association between the variant p53 Arg72Pro and the risk of oral cancer with HPV infection was detected in the Arg/Arg vs. Arg/Pro + Pro/Pro model [87]. The protein TP73 is structurally and functionally related to the p53 protein, and is involved in cell cycle regulation, induction of apoptosis has also been considered a tumor suppressor. The variant G4C14-to-A4T14 in the *TP73* gene has been reported to increase the risk of HPV-16-associated squamous cell carcinoma of the oropharynx (SCCOP) [88,89,90]. On the other hand, the SNPs *rs10900598, rs1380576* and *rs11801299* in the *MDM4* gene, which encodes a protein that acts as a negative regulator of p53, were significantly associated with HPV16+ SCCOP and when combined all risk genotypes of the three polymorphisms, the patients carrying 1–3 *MDM4* risk genotypes were approximately 2.5 times as likely to have an HPV16-positive tumor than those with no risk genotypes [91]. 

Another gene of interest is *TGFβ*, which encodes the transforming growth factor beta protein (TGFβ protein), an anti-inflammatory cytokine that is an important negative regulator of immune responses and has been implicated in promoting the persistence of HPV infection [2]. A study by Guan et al. found a positive association between patients with the variant T869C in the *TGFβ1* gene and having HPV16-positive HNSCC compared to patients without a variant in the *TGFβ* gene [92]. This variant also was associated with therapy response and survival outcomes in patients with HPV+ oropharyngeal cancer [93] and in a recent meta-analysis the risk association conferred by the T869C variant remained statistically significant, especially in Asian patients [94]. This suggests that individuals with variants in immune-related genes may be genetically predisposed to a higher risk of HPV-mediated HNSCC.

The first GWAS for discovering genetic factors associated with HNSCC was conducted in European patients with upper aerodigestive tract (UADT) cancers, including 2091 cancer cases (encompassing of the oral cavity, pharynx, larynx and esophagus) and 3513 controls, within the International Head and Neck Cancer Epidemiology (INHANCE) consortium and identified two novel variants significantly associated with susceptibility to UADT cancers, the variant 4q21 (*rs1494961*) located near *HEL308* and *FAM175A*, both of which encode proteins involved in DNA repair machinery, and the variant 12q24 (*rs4767364*) [95]. However, data from the subgroup stratification showing that despite the association of the *rs1494961* variant with cancer risk did not remain statistically significant in never-users of tobacco or alcohol and younger than 50 years of age, together with the fact that a considerable fraction of UADT cancers are not HPV+ HNSCC, preclude any definitive conclusions regarding the role of *rs1494961* in HNSCC susceptibility.

In a subsequent GWAS conducted in Greater Boston, Massachusetts that included 545 cases of confirmed HNSCC and 676 controls, the variant *rs4767364* in the *FLJ13089* gene was significantly associated with HNSCC. There was also a multiplicative interaction between the variant *rs1494961* (*HEL308* gene) and cigarette use of greater than 70 pack-years among HNSCC cases, implying that this genetic variant is more relevant to the high-risk population. Even though the HPV16 serologic status of case and control subjects was ascertained in this study with a greater prevalence of HPV16 seropositivity in cases than that in controls, unfortunately, the HPV positivity in tumor samples was not provided and was not included in subgroup stratification analysis [96], thus the exact relevance of these variants in HPV+ HNSCC is not well-defined. Of note, a validation study conducted in China (397 cancer cases and 900 controls), where six selected SNPs were directly genotyped using TaqMan allelic discrimination assay, revealed no association between the variant *rs1494961* and HNSCC susceptibility. Conversely, the variant *rs1229984* at 4q23 significantly increased the risk of HNSCC, while the variant rs671 at 12q24 significantly decreased the disease risk [17]. 

A large GWAS tested the association of genetic variations (296,728 SNPs) in numerous immune-related genes in 2091 UADT cancer patients (mainly head and neck cancer with esophageal cancers) and 8334 cancer-free controls. Variants in 14 immune-related genes, particularly those related to TGFβ signaling, were found to be associated with susceptibility to HPV-related but not HPV-unrelated HNSCC and this was findings were replicated in an independent dataset of cervical cancer. Further analysis highlighted a pivotal contribution of TGFβR1 containing p38–MAPK pathway to oropharyngeal cancer and cervical cancer and revealed TGFβR1 was overexpressed in oropharyngeal cancer, cervical cancer and HPV+ head and neck cancer tumors [97]. Even though this study was limited by the lack of gold-standard tumor HPV status and HPV positivity was assessed by serology to HPV16, this is the first study that exploited GWAS as a high-throughput strategy to examine immunogenetic susceptibility to HPV-associated HNSCC.

A GWAS of samples derived from patients with cancer in the oral cavity and pharynx (6034 cases and 6585 controls) from Europe, North America and South America; detected 8 loci, 7 of which are novel for these cancer sites. Oral and pharyngeal cancers combined were associated with loci at 6p21.32 (*rs3828805*, *HLA-DQB1*), 10q26.13 (*rs201982221*, *LHPP*) and 11p15.4 (*rs1453414*, *OR52N2/TRIM5*). Oral cancer was associated with two new regions 2p23.3 (*rs6547741*, *GPN1*) and 9q34.12 (*rs928674*, *LAMC3*), and with known cancer loci: 9p21.3 (*rs8181047*, *CDKN2B-AS1*) and 5p15.33 (*rs10462706*, *CLPTM1L*). Oropharyngeal cancer associations were limited to the HLA region and classical HLA allele imputation revealed a protective association with the class II haplotype DRB1*1301-DQA1*0103-DQB1*0603. A subgroup analysis showed that the genetic associations found in oropharyngeal cancer were considerably stronger in HPV-positive cancers compared to HPV-negative cancers [98].

A recent GWAS study consisting of 2171 cases and 4493 controls of non-Hispanic white and further validated in a cohort of oral and pharynx cancer (5205 cases and 3232 controls) of European ancestry) with squamous cell carcinoma of the head and neck (SCCHN). Four previously reported loci, 2p23.1, 5p15.33 (*rs1265081*, CCHCR1), 6p21.32 (*rs3135001*, *HLA-DQB1*) and 6p21.33(*rs13211972*, *MICA*) and two novel loci 6p22.1 (*rs259919*) and 18q22.2 (*rs142021700*) were associated with SCCHN risk, thus highlighting the importance of HLA loci for oropharyngeal cancer risk. The variant *rs259919* in the 6p22.1 region correlated with significantly lower levels of *ZFP57* transcripts in various tissues and an increased risk of SCCHN [99]. Importantly, ZFP57 is a Kruppel-associated box (KRAB) containing zinc-finger protein involved in transcriptional regulation and DNA methylation that is preferentially expressed early in development and has been proposed to act as a putative regulatory variant associated with cancer and HIV-1 infection [100]. Similarly, the variant *rs13211972* in the 6p21.33 region correlated with a significant decrease in mRNA expression levels of MICA and an increased risk of SCCHN [99] thus substantiating the relevance of MICA expression in carcinogenesis, including other virus-associated cancers, such as in EBV+ nasopharyngeal carcinoma [101] and hepatitis B virus (HBV)-induced hepatocellular carcinoma [102]. 

A recent meta-analysis of GWAS data of 61,961 controls and 13,887 cases of UADT cancers, including squamous cell carcinoma (SCC) of the lung, oropharyngeal region, larynx and esophagus in individuals of European ancestry identified a significant region within 2q33.1 (*rs56321285*, *TMEM273*) and 3 other suggestive regions at 1q32.1 (*rs12133735*, near *MDM4*), 5q31.2 (*rs13181561*, *TMEM173*) and 19p13.11 (*rs61494113*, *ABHD8*). Gene-based analyses also identify a list of SCC-related genes that are involved in DNA damage response and epigenetic regulation pathways [103]. Despite the HPV status was not systematically investigated in this study, these results suggest the possible existence of some overlap in the genetic factors influencing the risk of UADT cancers in European populations. Further studies are needed to delineate the precise role of genetic variation on the susceptibility to HNSCC. In addition, contrary to cervical cancer, where the majority of cases are linked to HPV infection, an important portion of HNSCC cases are not directly associated with HPV, therefore, care should be taken when analyzing the role of genetic factors identified by genetic association studies. 

## 6. Genetic Variants and HPV-Induced Anogenital Cancers

Persistent infection by oncogenic HPVs among men and women is responsible for the development of a considerable fraction of cancers of the anogenital tract and their precursor lesions. Epidemiological studies have shown that around 85% of the anal SCC that occur annually worldwide in men and nearly 50% of penile cancers are associated with HPV infection. In women, viral DNA has been detected in 40% of vulvar cancer cases and more than 90% of vaginal cancers [86,104,105,106]. Among HPV DNA positive anogenital cancer cases, HPV-16 is the most frequently found followed distantly by HPV-18 [86,107]. Given the rarity of anogenital cancers, only a few gene association studies have been conducted worldwide. We found no GWAS using samples from anogenital cancers and candidate gene association studies reported so far have focused on genes related to the immune response and especially on vulvar or vaginal cancers, which are relatively more common than penile or anal cancers. 

A population-based case-control study investigated the risk of cervical and vulvar cancer associated with common genetic variations in 560 tagging SNPs (tagSNPs) in candidate cytokine genes in the Th1 and Th17 pathways. The study included 399 invasive SCCs and 502 adenocarcinomas of the cervix; 357 in situ or invasive vulvar SCC; and 1109 controls from the Seattle area. The study observed a significantly increased risk of HPV-positive vulvar cancers associated with variant alleles in the *CSF2* (*rs25882* and *rs27438*) and *IL-12B* (*rs2569254* and *rs3181225*) genes. They also reported a significant association for the T allele in SNP *rs879576* (*IL17RA* gene) with an increased risk for HPV16-positive SCC of the cervix [108]. Thus, this study provided evidence that common genetic variation in the immune response pathways, particularly Th1 and Th17, may influence individual susceptibility to the development and progression of HPV-related cervical and vulvar carcinomas. Using a similar approach, a population-based case-control study (886 cervical cases, 517 vulvar cases and 1100 controls), genotyped a total of 17 tagSNPs in the *CD83* gene (chromosome 6p23), whose product (CD83 protein) is expressed on the surface of mature dendritic cells and plays important roles in the immune response against HPV). In this study, whereas tagSNPs in the CD83 region were not associated with risk of either cervical or vulvar cancer, the variant rs853360 was associated with a decreased risk of cervical SCC [109]. Of note, four SNPs analyzed in this study (rs9296925, rs853360, rs9370729 and rs750749) were significantly associated with the risk of invasive cervical cancer in patients harboring HPV16/18 subtypes as reported in an earlier study [110]. 

Another study extended the analysis to 205 tagSNPs in and around 32 candidate gene regions within the *TLR, TNF* and the nuclear factor κB (*NF-κB*) signaling pathways, which are involved in the host immune response against HPV infection [2]. SNPs within the TNF region were significantly associated with the risks of vulvar cancer and cervical cancer. In particular, the G allele of *rs2256965* (*LST1* gene), was significantly associated with a 40% increased risk of vulvar cancer, but to a lesser extent with cervical cancer. In addition, the SNP *rs2239704* (allele A) in the 5′ UTR of the *LTA* gene was significantly associated with increased risks of both cervical cancer and vulvar cancer [111], however, a previous study conducted in Costa Rica reported no association between the *rs2239704* variant and risk of cervical cancer [57]. 

The cytokine IL-12, produced primarily by antigen-presenting cells, stimulates CD4+ T cells and NK cells to differentiate toward the Th1 phenotype and is implicated in host immune response against viruses, including HPV. In a case-control study, using 76 tagSNPs from seven candidate genes (*IL-10, IL-12A, IL-12B, IL-10RA, IL-10RB, IL-12RB1* and *IL12RB2*), genetic variants in the IL12B gene, (*rs3181225, rs3181224, rs3212227*) were associated with risk of cervical and vulvar cancers. In particular, the minor allele of the *IL12B rs3181224* 3′ UTR SNP was associated with a reduced risk of vulvar SCC [112].

Penile squamous cell carcinoma (PSCC) is a rare but often aggressive disease (~ 22,000 cases per year) with a higher incidence is in less developed countries, where penile cancer can account for up to 10% of cancers among men in some parts of Africa, South America and Asia [113]. Due to the rarity of this disease, data on gene association studies are scarce, with most of the published studies enrolling less than 100 cases. The variant p.Arg72Pro (*rs1042522*) in the *TP53* gene has been investigated in several cancer types with conflicting results [114], and although several studies suggest that this variant is a risk factor of HPV-associated cancers [87,115], a pooled analysis of individual data on 7946 cases and 7888 controls from 49 different studies worldwide found no association between cervical cancer and *TP53* codon 72 polymorphisms when the analysis was restricted to methodologically sound studies and subgroup analyses (based on infection with HPV, ethnic origin, Hardy-Weinberg equilibrium and study quality) indicated that excess risks were most likely due to errors in study methods rather than due to clinical or biological factors [116]. However, in an updated meta-analysis of 49 publications (7269 patients with invasive cervical cancer or premalignant cervical lesions, and 5326 healthy controls) the Arg variant of p53 Arg72Pro was associated with progression of squamous intraepithelial lesions (SIL) to cervical cancer only in the presence of HPV positivity [117]. 

In our literature search, we found three studies conducted in three different populations testing a potential association between p.Arg72Pro variant with PSCC, however, these studies reported negative findings, namely the variant p.Arg72Pro does not represent a risk factor for the development of in PSCC [118,119,120]. 

A small study from UK Caucasian women, including vulvar intraepithelial neoplasia (VIN) and invasive vulvar squamous cell carcinoma (VSCC) reported a lower frequency of arginine homozygotes (p.Arg72) in both the vulvar cancer group (42%) and the VIN group (29%) compared with the control group (63%) [121]. Another UK study that included 72 adult Caucasian men with a clinical and histological diagnosis of penile intraepithelial neoplasia (PeIN) revealed that HLA-C*15 confers susceptibility to PeIN, whereas HLA-DQA1*01 protects against PeIN. In this study, *Alphapapillomavirus* types were detected in 85% of cases and HPV16 was the most prevalent genotype (48.6%) followed by HPV33 (9.7%); with multiple infections documented in 25% of cases. Notably, HPV16-associated PeIN cases showed no statistically significant association with HLA genotype after multiple corrections [122]. 

In Vietnamese patients with HPV+ anogenital cancers, our group reported an association between the allele LNK (*rs1049174*) in the *NKG2D* gene with increased cancer susceptibility, and these results were verified in a group of women with cervical cancer. In functional studies, NK cells from individuals harboring the LNK genotype expressed lower levels of the NKG2D receptor and displayed a reduced cytotoxic activity against target cells, compared with NK cells with the HNK genotype. This was in part, due to a higher affinity of the LNK allele to miR-1245 [123], which is a negative regulator of NKG2D expression [124]. Since the NKG2D receptor play a crucial role in cancer immunosurveillance [77,125], individuals with the LNK allele appear to have a relatively higher risk of developing various malignancies [101,126,127], including HPV-induced cancers. 

## 7. Concluding Remarks and Future Directions

HPV infections are common worldwide, with an estimated lifetime incidence exceeding 70% and although most infections are transient and cleared through an effective host immune response, susceptible individuals carrying oncogenic HR-HPV subtypes may develop a persistent infection, which can result (depending on the infected body niche) in the development of cervical cancer, anogenital cancers and a growing fraction of HNSCCs. 

Population-based studies and heritability estimates focused on cervical cancer (the most common and most studied HPV-associated cancer) suggest an inherited genetic contribution to disease risk of around 30%, thus substantiating the contribution of the genetic compartment to disease susceptibility. Over the last three decades, numerous gene association studies, using either the candidate gene approach or GWAS, have been conducted across different populations, in an attempt to identify genetic factors associated with the development of HPV-associated cancers, as well as potential genetic determinants of persistent infection with HPV. 

Multiple and diverse candidate gene studies have reported SNPs in genes associated with the immune response, apoptosis, DNA repair, as well as within the HLA region, which appear to predict susceptibility to persistent HPV infection and HPV-induced cancers, however, due to study heterogeneity and the small sample size, the replication of findings has been a frequent limitation of those studies. Similarly, a growing number of GWASs of HPV-associated cancers have been reported in recent years. These studies varied in size and type of specimen analyzed and the replications of results in different ethnic groups have been limited by the modest sample sizes and disease heterogeneity (inclusion of patients with invasive cancer or premalignant lesions) of some of these studies. However, the most consistently identified allelic variation is at the 6p21.3 locus, within the HLA region, as confirmed across multiple populations of European and Asian descendants. A recent GWAS has confirmed the importance of HLA genes for the genetic susceptibility to cervical cancer, along with new risk variants in the *PAX8* and *CLPTM1L* genes, suggesting a disruption in apoptotic and immune function pathways plays a key role in the susceptibility to HPV-associated cancers. 

Future studies utilizing new generation technologies capable of analyzing massive amounts of data such as machine learning and other artificial intelligence tools will contribute to integrating host genetic, epigenetic variation, microbiome data along with viral genetics to further elucidate complex host–viral interactions to unravel the precise influence of host factors in the susceptibility to HPV-associated cancers. 

As mentioned above, SNPs in miRNAs or in miRNAs binding sites can alter individual susceptibility to HPV-associated cancers and HPV oncogenes, especially E6 and E7 are capable of modifying the expression of several miRNAs thus modulating gene expression [128]. In addition to miRNAs, other epigenetic regulators of gene expression, including DNA methylation and histone modifications, can be exploited by HR-HPVs to evade the host immune response or to promote malignant transformation. For example HR-HPVs alter host DNA methylome leading to the downregulation of key host genes, which was elicited by high-risk HPV E7 oncogene. In particular, genes encoding key proteins involved in the host immune response, such as HLA-E, were hypermethylated [129]. The epigenetic downregulation of HLA-E may contribute to virus immune evasion and persistent infection because HLA-E plays an important role in antiviral immunity by activating NK cells and CD8+ T cells. This implies that inhibitors of DNA methylation such as 5-aza-2-deoxycytidine and Histone deacetylase (HDAC) inhibitors, alone or in combination with chemotherapy or immunotherapy regimens may have therapeutic potential in HPV-induced cancers.

There are currently three prophylactic HPV vaccines on the market, the quadrivalent HPV vaccine (HPV 6, 11, 16 and 18), the bivalent vaccine (HPV 16 and 18) and the nonavalent vaccine (HPV 6, 11, 16, 18, 31, 33, 45, 52 and 58). Randomized clinical trials and real-world data have shown HPV vaccines to be highly efficacious to prevent genital warts and high grade squamous intraepithelial (HSIL) lesions of the cervix, vulva and vagina, although vaccine efficacy was lower in mid-adult (aged 24–45 years) women [130,131,132]. Previous studies have suggested the influence of genetic regulators on vaccine-induced immunity. For example, HLA class I and HLA class II genotypes and SNPs in cytokine/cytokine receptor genes (*IL12B, IL12RB1, IL2, IL10*) and the cell surface measles virus receptor the *CD46* gene, have been reported to affect measles vaccine-induced immunity [133]. To the best of our knowledge, it is currently unknown if genetic variants affect the individual response to HPV vaccines and there are no healthcare approaches to measure HPV vaccine-induced immunity in order to identify individuals who would benefit from vaccine boosters. Therefore, it is important to identify genetic markers of antibody response and long-term HPV immunity. In this regard, the use of personalized HPV vaccine designs based on individualized genetic risk, common host molecular genetic variations and HPV vaccine POI in different populations have been proposed recently [134]. 

## Figures and Tables

**Table 1:** Summary of GWAS on HPV+ cervical cancer.

1st Author, Year, Reference	Discovery Cohort	Confirmatory Cohort	Ethnicity	Number of SNPs Tested	Main Findings
Bowden S. Lancet Oncol. 2021 [14].	273,377 women (4769 CIN3 and invasive cervical cancer case samples and 145,545 control)	128,123 women (1648 invasive cervical cancer cases) from a Finnish dataset	Discovery cohort: UK (UK Biobank) Confirmatory cohort: Finland (FinnGen).	9 million SNPs	Six loci strongly associated with CIN3 and invasive cervical cancer. Two novel SNPs, *rs10175462 (PAX8)* and *rs27069 (CLPTM1L)* Verified previously reported SNPs: *rs9272050 (HLA-DQA1), rs6938453 (MICA), rs55986091 (HLA-DQB1)* and *rs9266183 (HLA-B).* Three SNPs, *rs10175462 (PAX8 gene), rs27069 CLPTM1L)* and *rs9272050 (HLA-DQA1)* were replicated in the confirmatory Finnish dataset.
Takeuchi F. Hum Mol Genet. 2019 [79]	2690 cases and 4712 controls (1289 cases and 2718 controls from previous Chinese study)	1320 cases and 1994 controls from newly reported Japanese study	Japanese GWAS study: East Asian populations	4,602,429 SNPs	- two new studies corroborated two previously reported loci in association with cervical cancer and identified significant associations at 5q14 with SNP *rs59661306* and at 7p11 with the SNP *rs7457728* - in 5q14 the chromatin region of the GWAS-significant SNPs was found to be in contact with the promoter of the *ARRDC3* gene - functional studies showed that *ARRDC3* knockdown in the HPV+ HeLa cells resulted in substantial reductions in cell growth and decreased susceptibility to HPV16 pseudovirion infection
Chen D. J. Natl. Cancer Inst. 2013 [18]	GWAS in women of European-ancestry with 1075 cervical cancer cases, and 4014 controls	replicated in 1140 case subjects and 1058 control subjects	European descendants	731,422 SNPs	- three independent loci in the MHC region at 6p21.3 were identified in association with cervical cancer - women carrying frameshift mutation of MICA gene have lower levels of membrane-bound MICA
Leo PJ. PLoS Genet. 2017 [41]	2866 cervical cancer cases and 6481 controls	Nd	European descendants	10,863,230 SNPs	- Identified risk and protective HLA haplotypes that are determined by the amino-acids carried at positions 13 and 71 in pocket 4 of HLA-DRB1 and position 156 in HLA-B. - host genetics is a major determinant of HPV-associated cervical cancer, with 36% of liability due to common genetic variants in the population.
Chen D. Oncotarget. 2016 [78]	analysis of data from GWAS of women of European ancestry (1075 cervical cancer cases and 4014 controls + replicated in 1140 case subjects and 1058 control subjects)	961 patients (827 with CIN3 and 123 with cervical cancer) and 1725 controls	European	5,471,179 SNPs	- provided support for previously identified loci at 6p21.3 - confirmed associations with reported HLA alleles - identified and replicated an independent signal at *rs73730372* at 6p21.3 in association with CIN3
Shi Y. Nat. Genet. 2013 [79]	1364 women with cervical cancer and 3028 female controls	Dataset 1: 1824 cases and 3808 controls. Dataset 2: 2343 cases and 3388 controls	Han Chinese population	563,339 SNPs	- identified strong evidence of associations between cervical cancer and two new loci: 4q12 (*rs13117307*) and 17q12 (*rs8067378*) - replicated an association between *HLA-DPB1* and *HLA-DPB2 (HLA-DPB1/2*) at 6p21.32 and cervical cancer (*rs4282438*)
Miura K. J. Med Virology. 2014 [84]	226 women with cervical cancer and 186 controls	Not included	Japanese	556,045 SNPs	- no statistically significant associations were found between the tested SNPs and susceptibility to cervical cancer

Abbreviations: SNP: single nucleotide polymorphism; HPV: human papillomavirus; nd: no data; CIN3: Cervical intra-epithelial neoplasia grade 3; MICA: MHC Class I Polypeptide-Related Sequence A; MHC: major histocompatibility complex; PAX8: Paired Box 8; ARRDC3: Arrestin Domain Containing 3; CLPTM1L: Cleft lip and palate transmembrane protein 1-like protein; GWAS: Genome-wide association study.

**Table 2:** Summary of GWAS on HPV+ head and neck cancer.

1st Author, Year, Reference	No. of Cases	No. of Controls	Ethnicity	No. of SNPs Tested	Gene Variants	Main Findings
Mckay JD, Plos Genet. 2011 [95]	2091 (oral cavity, pharynx, larynx and esophagus)	3513	European	nd	*rs1494961*, *rs4767364*	- two novel variants were associated with susceptibility to UADT cancers (q421 and *FAM175A*) - these variants encode proteins involved in DNA repair machinery - no statistical significance in those who never used alcohol, tobacco or were younger than 50 - no definitive conclusions on the role of 4q21 in HNSCC susceptibility
Liang C. Head Neck, 2012 [96]	545 (HNSCC)	676	none specified, conducted in Greater Boston, Massachusetts	nd	*rs4767364, rs1494961*	- the variant *rs4767464* in the *FLJ13089* gene was significantly associated with HNSCC - the genetic variation in the *HEL308* gene is more relevant to high risk populations
Levovitz C. Cancer Res. 2014 [97]	2091 UADT cancer patients (HNSCC)	8334	European	296,728	variants in 14 immune-related genes, TGFβR1	- variants in genes related to transforming growth factor beta signaling were found to be associated with susceptibility to HPV-related HNSCC - TGFβR1 was overexpressed in oropharyngeal, cervical and HPV+ Head and neck cancer tumors
Lesseur C. Nat Genet. 2016 [98]	6034 (oral cavity, pharyngeal cancer)	6585	Europe, North America and South America	nd	*rs3828805, rs201982221, rs1453414, rs6547741, rs928674, rs8181047, rs10462706*	- A GWAS of oral cavity and pharyngeal cancer detected 8 loci, with 7 being novel for these cancer sites - oral cancer associated with two new region - oropharyngeal cancer associations were limited to the HLA region - HLA allele imputation revealed a protective association with the class II haplotype - associations of oropharyngeal cancer was considerably stronger in HPV+ cancers compared to HPV- cancers
Shete S. Cancer Res. 2020 [99]	2171 GWAS, 5205 cohort	4493 GWAS, 3232 cohort	European	nd	*rs1265081, rs3135001, rs13211972, rs259919, rs142021700,*	- four previously reported loci, and two novel loci were associated with SCCHN risk (highlighting the importance of HLA loci for oropharyngeal cancer risk) - the variant *rs259919* in the 6p22.1 region correlated with significantly lower levels of ZFP57 transcripts in various tissues and an increased risk of SCCHN

Abbreviations: SNP: single nucleotide polymorphism; HPV: human papillomavirus; nd: no data; SCCHN: Squamous cell carcinoma of head and neck; UADT: upper aero-digestive tract; FAM175: FAMILY WITH SEQUENCE SIMILARITY 175. This gene also known as abraxas 1 or BRCA1 A complex subunit; GWAS: Genome-wide association study.

## Data Availability

Not applicable.
